# Supplementary material for: Oxycodone self-administration activates the mitogen-activated protein kinase/ mitogen- and stress-activated protein kinase (MAPK-MSK) signaling pathway in the rat dorsal striatum
Source: Sci Rep. 2021 Jan 28;11:2567. doi: 10.1038/s41598-021-82206-3 (PMC7843984; doi:10.1038/s41598-021-82206-3)
Supplement: Supplementary file 1 — Supplementary Information. [file 41598_2021_82206_MOESM1_ESM.pdf]

Supplementary Information

Oxycodone self-administration activates the mitogen-activated protein kinase/ mitogen- and stress-activated protein kinase (MAPK-MSK) signaling pathway in the rat dorsal striatum

Christopher A. Blackwood, Michael T. McCoy, Bruce Ladenheim, and Jean Lud Cadet

Supplementary Figure S1. Effects of oxycodone SA on PKC phosphorylation. (A) Images of western blot and **(B, C)** quantification of PKC and pPKC. **(B)** PKC protein levels were not significantly affected in any group. **(C)** pPKC abundance is increased in only LgA-H rats. **(D)** pPKC/PKC ratios are increased in only the LgA-H rats. **(E)** pPKC/PKC ratios correlate with amount of oxycodone taken during the experiment (n=5-6 Sal; n=6 ShA; n=5 LgA-L; n=6 LgA-H). Key to statistics: *, ** = $p < 0.05$, 0.01, respectively, in comparison to Sal rats; #, ## = $p < 0.05$, 0.01, respectively, in comparison to ShA rats; $ = $p < 0.05$ in comparison to LgA-L rats. Statistical analyses were performed by one-way ANOVA followed by Bonferroni or Fisher's PLSD post hoc test. The correlation coefficients and p values are shown on the graph.

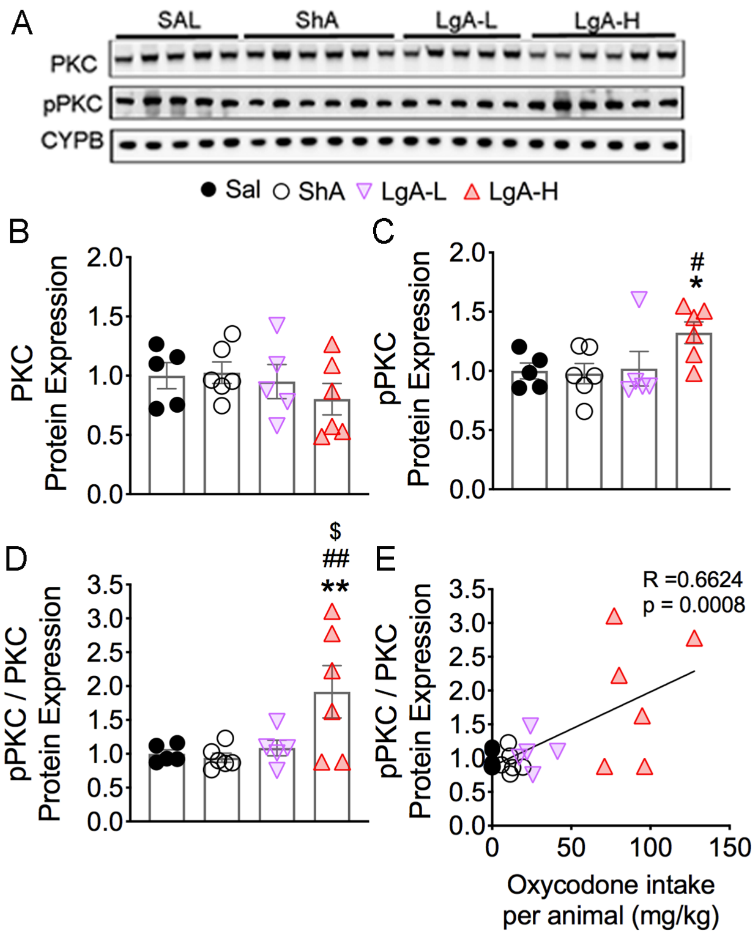

Supplementary Figure S2. Exposure to oxycodone SA shows no changes in PKA. (A) Images of western blot and quantification of **(B)** PKA and **(C)** pPKA protein expression. Protein levels of **(B-D)** PKA, pPKA, and pPKA / PKA ratios show no significant changes. Similarly, **(E)** ratio of pPKA / PKA showed no correlation to the amount of oxycodone taken (n=6 Sal; n=6 ShA; n=6 LgA-L; n=6 LgA-H). Full-length blots are presented in Supplementary Figure S5. Statistical analyses were performed by one-way ANOVA followed by Bonferroni or Fisher's PLSD post hoc test. The correlation coefficients and p values are shown on the graph.

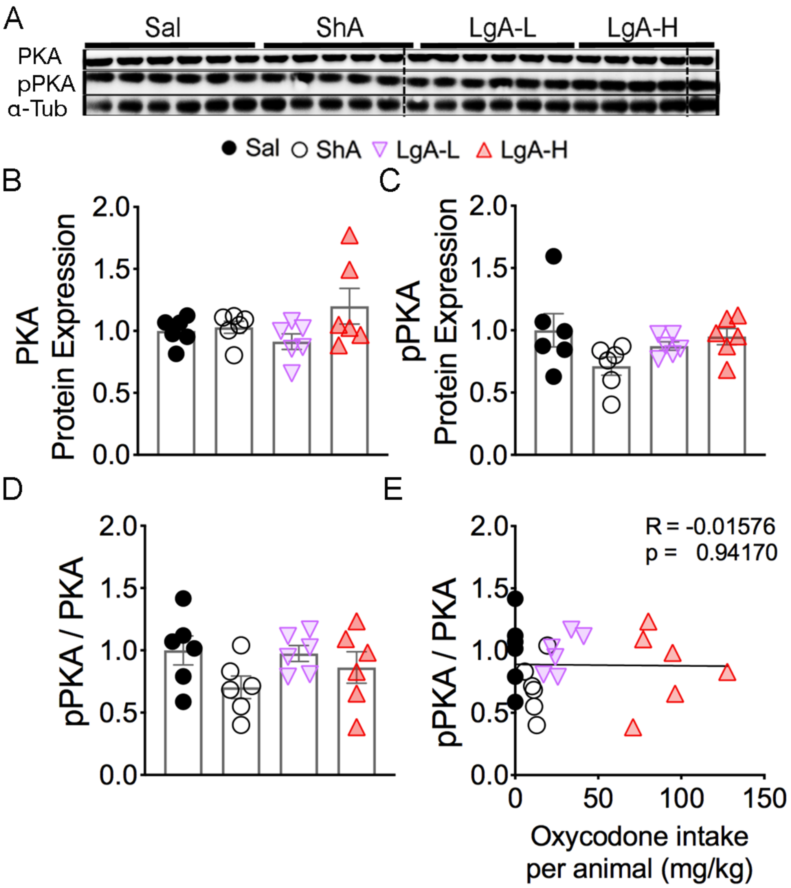

Supplementary Figure S3. Exposure to oxycodone SA increases Tip60. (A) Images of western blot and **(B)** quantification of Tip60 protein levels show increases in all drug groups (n=5 Sal; n=6 ShA; n=6 LgA-L; n=6 LgA-H). Key to statistics: *, **= p < 0.05, 0.01, respectively, in comparison to Sal rats. Stats were performed by one-way ANOVA followed by Bonferroni or Fisher's PLSD post hoc test.

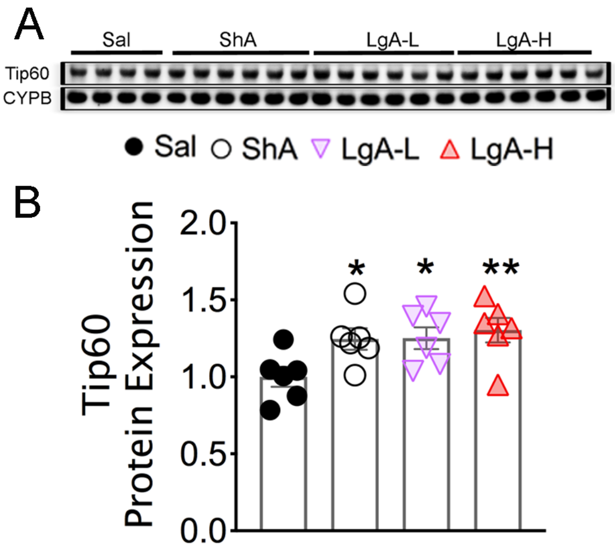

Supplementary Figure S4. mRNA expression on the members of Ncoa family after exposure to oxycodone SA and early withdrawal. The mRNA levels of **(A)** *Ncoa1*, **(B)** *Ncoa2*, and **(C)** *Ncoa3* show significant increases in the LgA-H group. The mRNA levels of *Ncoa1*, *Ncoa2*, and *Ncoa3* show a positive correlation with **(D-F)** pMSK1, **(G-I)** pMSK2, and **(J-L)** H3S10pK14Ac. However, the mRNA expression of *Ncoa1, Ncoa2, and Ncoa3* shows no correlation **(M-O)** CBP protein expression (n=4-9 Sal; n=5-9 ShA; n=4-8 LgA-L; n=5-9 LgA-H). Key to statistics: **, *** = p < 0.01, 0.001, respectively, in comparison to Sal rats; #, ### = p < 0.05, 0.001, respectively, in comparison to SHA rats; $$, $$$ = p < 0.01, 0.001, respectively, in comparison to LgA-L rats. Statistical analyses are as described in Fig. 2.

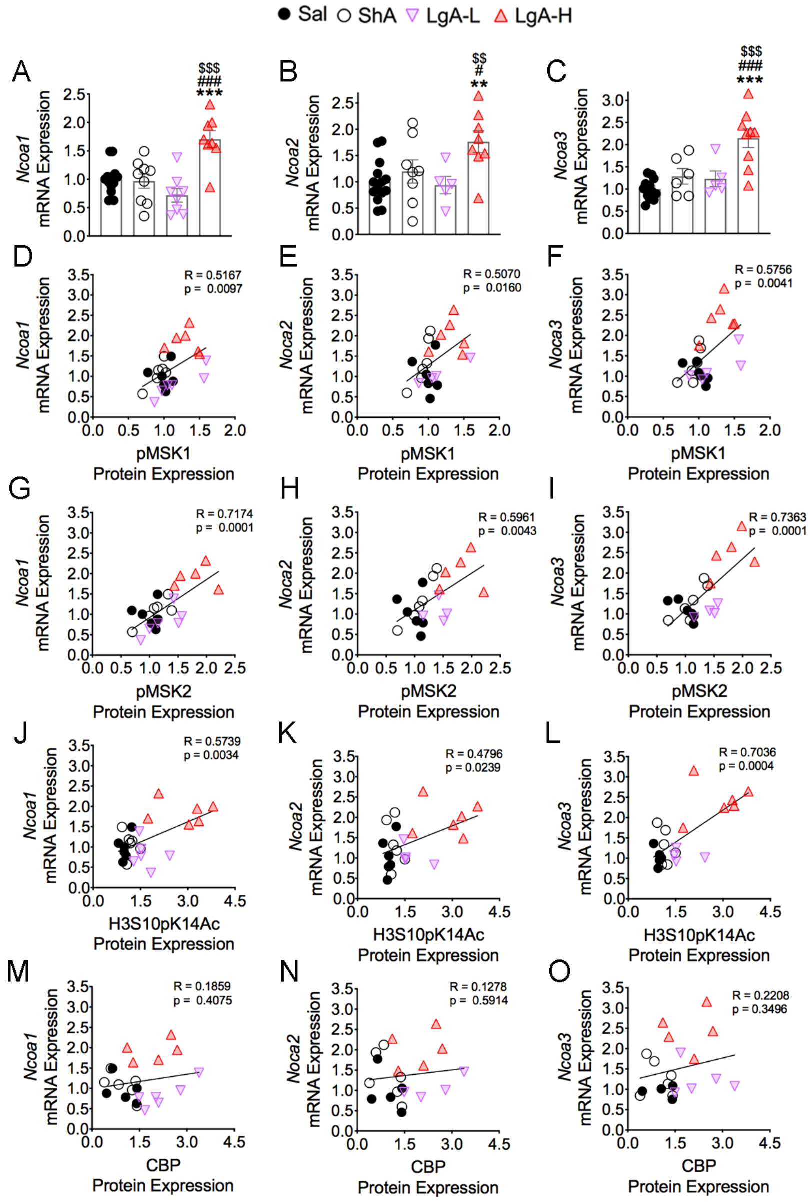

Supplementary Figure S5: Full length blots images of

Figure S1: PKC, pPKC, CYPB

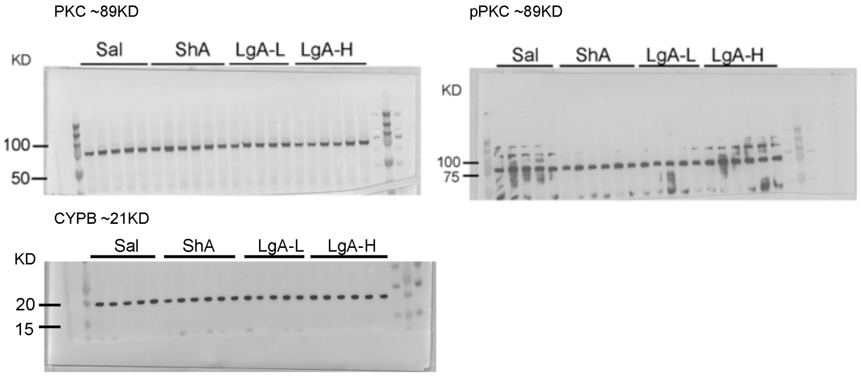

Figure S2: PKA, pPKA, Alpha-Tubulin

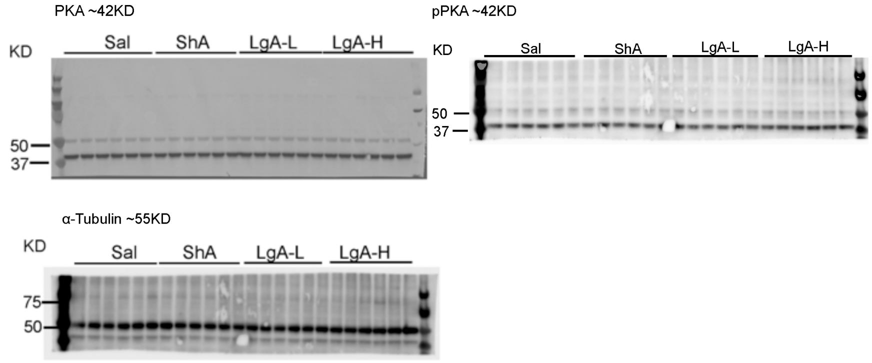

Figure 2: ERK1/2, pERK1/2, CYPB

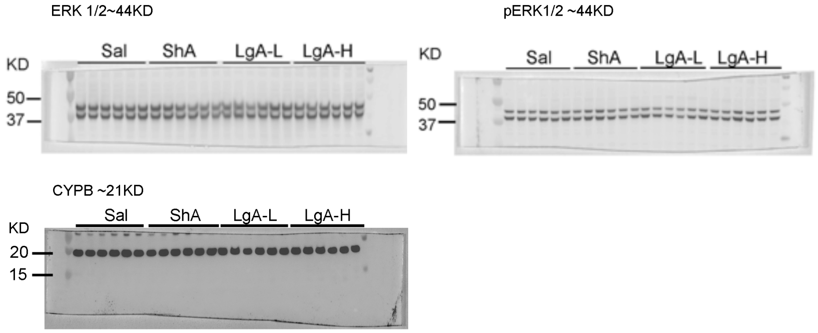

Figure 3: MSK1, pMSK1, MSK2, pMSK2, CYPB(s). Black arrow indicates bands of interest.

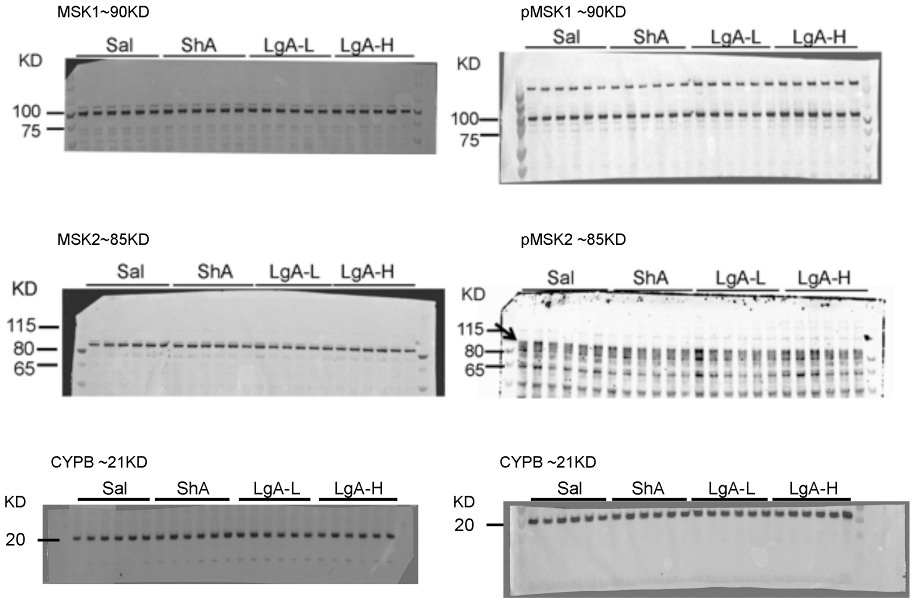

Figure 4: CREB, pCREB, CYPB(s). Black arrow indicates bands of interest.

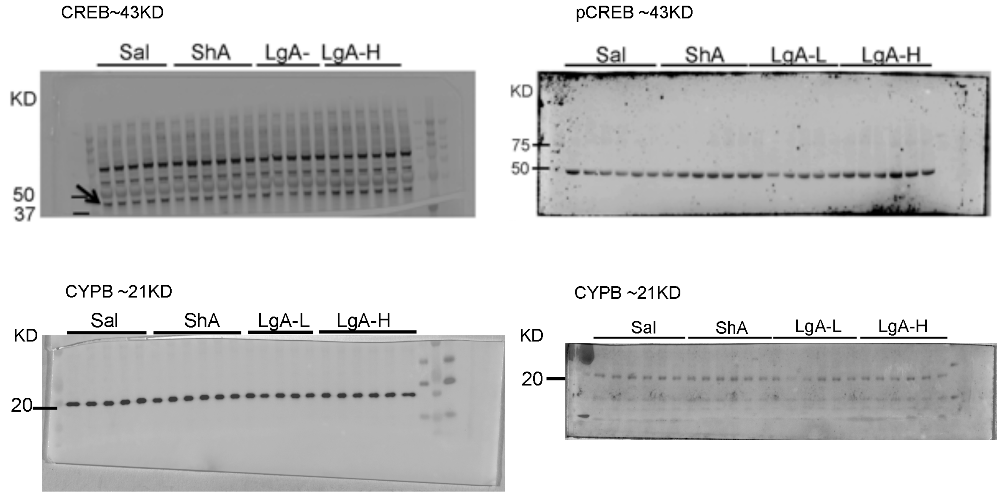

Figure 5: H3, H3S10pK14Ac, CYPB. Black arrow indicates bands of interest.

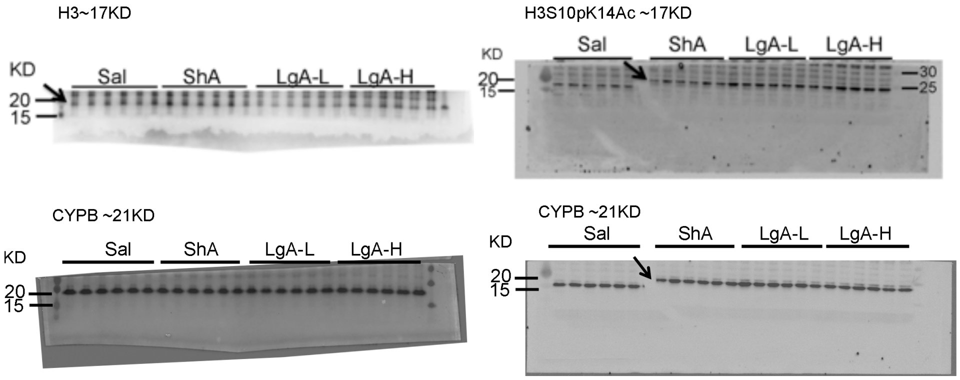

Figure 6: CBP, H3K27Ac, CYPB, alpha-Tubulin

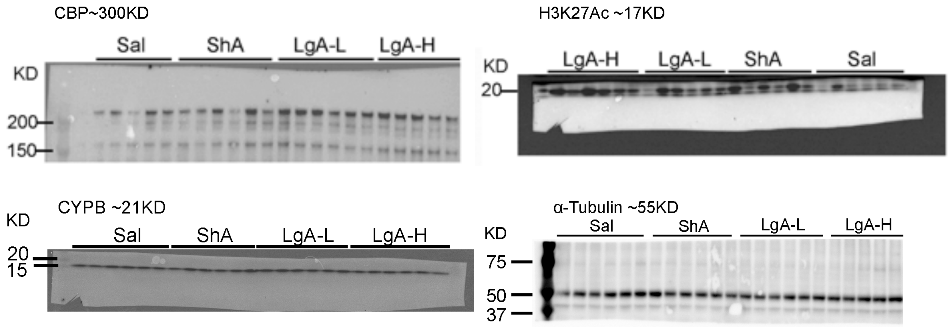

Figure S3: Tip60, CYPB

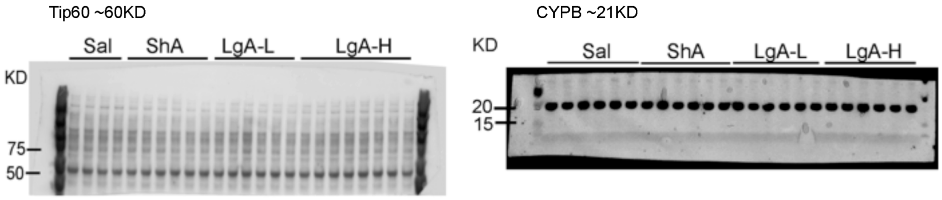

Supplementary Table S1. Antibody list

Symbol	Source	Dilution	Catalog #	RRID	Company
PKC	Rabbit	1:1,000	2056	AB2284227	Cell Signaling, Danvers, MA, USA
pPKC	Mouse	1:1,000	SC-377565	AB10611031	Santa Cruz Bio. Tech., Dallas, TX, USA
ERK1/2	Rabbit	1:1,000	06-182	AB310068	Sigma-Aldrich, St. Louis, MO, USA
pERK1/2	Rabbit	1:1,000	4370	AB2315112	Cell Signaling, Danvers, MA, USA
MSK1	Rabbit	1:1,000	3489	AB2285349	Cell Signaling, Danvers, MA, USA
pMSK1	Rabbit	1:1,000	9595	AB2181783	Cell Signaling, Danvers, MA, USA
MSK2	Rabbit	1:10,000	3679	AB2181641	Cell Signaling, Danvers, MA, USA
pMSK2	Rabbit	1:10,000	SAB4504629	AB11184854	Sigma-Aldrich, St. Louis, MO, USA
CREB	Rabbit	1:1,000	9197	AB331277	Cell Signaling, Danvers, MA, USA
pCREB	Mouse	1:1,000	9196	AB331275	Cell Signaling, Danvers, MA, USA
Histone H3	Rabbit	1:10,000	06-755	AB2118461	Sigma-Aldrich, St. Louis, MO, USA
H3S10pK14Ac	Rabbit	1:1,000	07-081	AB310366	Sigma-Aldrich, St. Louis, MO, USA
H3K27Ac	Rabbit	1:1,000	39034	AB2722569	Active Motif, Carlsbad, CA, USA
CBP	Rabbit	1:500	7389	aB2616020	Cell Signaling, Danvers, MA, USA
PKA	Mouse	1:1,000	4782	AB2170170	Cell Signaling, Danvers, MA, USA
pPKA	Rabbit	1:1,000	5661	AB10707163	Cell Signaling, Danvers, MA, USA
TIP60	Rabbit	1:10,000	MABE430	AB2551948	Sigma-Aldrich, St. Louis, MO, USA
CYPB	Rabbit	1:10,000	ab16045	AB443295	Abcam, Cambridge, MA, USA
Alpha-Tubulin	Mouse	1:25,000	T6074	AB477582	Sigma-Aldrich, St. Louis, MO, USA
HRP (rabbit)	Goat	1:5,000	7074	AB2099233	Cell Signaling, Danvers, MA, USA
HRP (mouse)	Horse	1:5,000	7076	AB330924	Cell Signaling, Danvers, MA, USA

Supplementary Table S2. List of RT-qPCR primers

Gene Name	Forward	Reverse
Aldh1a7	CTT GGA TAG TGC TGT TGA GTT T	TTT CCT AGA ACG TAT TTC TTA GCC C
Angp14	AGC AAC TGT TCC AGA AGG	CAA TGA GCT GGG CCA TC
Brd2	CTC ATG TTC TCC AAC TGC TAT AA	CAA GGC AGT AGA GAC AGG TA
Brd3	CAC TCT GGA AAC ATC AGT TTG	TAA ACA TGG TGT TGA AGT CCT
Cacna2d3	GAT GAA TGG TCC TAC TGC AAC A	TCC AAT AGG CTT CGA TAG GG
Dio2	CTT GCT GAT CAC TCT TCA GAT T	TTA ACC TGT TTG TAG GCG TC
Dusp14	GGA TCC TTC GAA CGC TAG CTC	TCC TGG ACG TAC TCG CAA AC
GluA1	GGC AAA TAC GCC TAC C	ACT CGA TTA AGG CAA CC
GluA2	TCC TAC ACG GCT AAC TT	GCT CGA TGT ACT CGT TC
Itpka	GTA GTA CAT TAG GAG CTG GC	GTA CAA TTC TGG ATC GGG AC
Kcnc4	CCT AAG ACA TGG TCA GGA AT	GTG GTA CAT GCT TCT TTC GTT
Kcng1	TGT TCC CAA ATG TGC CAC AA	CAG CAG TGT GAC GTA GTA AG
Ncoa1	GGT ACT TGT CCC TCT TCC CA	GTC CAG ACA CTG ACA TAG CA
Ncoa2	AGG AAG AAC TGA TGA ACA AGA G	CAT CCG ACA GTT GAA GGT ATG
Ncoa3	TGT ATC GTT TCT CAT TGG CTG	GAT TCG GGT TTG GTC TAC AT
Nos1	CCC AAG TTC GAC TGG TTT AAG	GTA TCG AGA GTT GTC ACA GTA G
Pdgfd	CAA GGA TAA CTT CAA GAA CAA ACC A	AGA ACT TGT GAC TGA TTC CC
Pdgfrl	CGG AAC AGA CAT TGT GTA CG	CGC TGG GAA CCT CTA CAT AA
Pdk4	GAT TAC TGA CCG CCT CTT TAG	ATG GAA TAG AGA TTC AGA TCT CCT
Prph	AGG TTA GAA GAA GAA ACT CGC	GTA GCT TCT TGA GGA ACT CAA T
Rtp2	GGA ACC AGA GCC TTA GGT TA	GCC ACC TCC ATC TTC TCA TA
Wnt4	CAG TGG AGA TCT GGA GAA GT	GTT GTT GTG AAG GTT CAT GAG T
